# The Molecular Epidemiological Characteristics and Genetic Diversity of *Salmonella* Typhimurium in Guangdong, China, 2007–2011

**DOI:** 10.1371/journal.pone.0113145

**Published:** 2014-11-07

**Authors:** Jiufeng Sun, Bixia Ke, Yanhui Huang, Dongmei He, Xiaocui Li, Zhaoming Liang, Changwen Ke

**Affiliations:** 1 Guangdong Provincial Institute of Public Health, Guangdong Provincial Center for Disease Control and Prevention, Guangzhou, China; 2 Institute of Microbiology, Guangdong Provincial Center for Disease Control and Prevention, Guangzhou, China; 3 Department of Epidemiology, School of Public Health and Tropical Medicine, Southern Medical University, Guangzhou, China; University of Birmingham, United Kingdom

## Abstract

**Background:**

*Salmonella enterica* serovar Typhimurium is the most important serovar associated with human salmonellosis worldwide. Here we aimed to explore the molecular epidemiology and genetic characteristics of this serovar in Guangdong, China.

**Methodology:**

We evaluated the molecular epidemiology and genetic characteristics of 294 endemic *Salmonella* Typhimurium clinical isolates which were collected from 1977 to 2011 in Guangdong, China, and compared them with a global set of isolates of this serovar using epidemiological data and Multilocus Sequence Typing (MLST) analysis.

**Principal Finding:**

The 294 isolates were assigned to 13 Sequencing types (STs) by MLST, of which ST34 and ST19 were the most common in Guangdong. All the STs were further assigned to two eBurst Groups, eBG1 and eBG138. The eBG1 was the major group endemic in Guangdong. Nucleotide and amino acid variability were comparable for all seven MLST loci. Tajima’s D test suggested positive selection in *hisD* and *thrA* genes (p<0.01), but positive selection was rejected for the five other genes (p>0.05). In addition, The Tajima’s D test within each eBG using the global set of isolates showed positive selection in eBG1 and eBG138 (p<0.05), but was rejected in eBG243 (p>0.05). We also analyzed the phylogenetic structure of *Salmonella* Typhimurium from worldwide sources and found that certain STs are geographically restricted. ACSSuT was the predominant multidrug resistance pattern for this serovar. The resistant profiles ACSSuTTmNaG, ACSSuTTmNa and ACSuTTmNaG seem to be specific for ST34, and ASSuTNa for ST19.

**Conclusion:**

Here we presented a genotypic characterization of *Salmonella* Typhimurium isolates using MLST and found two major STs are endemic in Guangdong. Our analyses indicate that genetic selection may have shaped the *Salmonella* Typhimurium populations. However, further evaluation with additional isolates from various sources will be essential to reveal the scope of the epidemiological characteristics of *Salmonella* Typhimurium in Guangdong, China.

## Introduction

*Salmonella enterica* is one of the leading causes of zoonotic food-borne disease worldwide [1], [2]. The global burden of disease caused by *Salmonella* infections is substantial, with more than 90 million human cases per year and costs of over $7 billion annually in Europe and the USA [2], [3]. *Salmonella* Typhimurium, one of ∼2500 serovars of *Salmonella enterica*, has been isolated in many geographically diverse regions and has caused sporadic outbreaks [3], [4], [5]. *Salmonella* Typhimurium is the most common cause of salmonellosis in Africa and North America [1], whereas in Europe, it is the second most common endemic serovar isolated from humans [5], [6].

In Asia, multiple epidemiological studies have addressed the characteristics of *Salmonella* Typhimurium in Thailand, Japan, Hongkong and Taiwan [7], [8], [9]. However, the incidence of *Salmonella* infections on the mainland of China has not been well documented. Laboratory-based surveillance of nontyphoidal *Salmonella* infections was initiated in China in 2008 [10], and the results showed that serotypes Enteritidis (12.5%) and Typhimurium (45.2%) were frequently isolated. Studies in Henan province in China also showed that *Salmonella* Typhimurium was predominant [11]. Our previous study of 1,764 *Salmonella enterica* isolates from 128 serovars in Guangdong province demonstrated that during 2007 to 2012, *Salmonella* Typhimurium (n = 523, 29.65%) was one of the most common serovars causing infant salmonellosis [12]. In addition, we found a major serovar shift from *Salmonella* Enteritidis to *Salmonella* Typhimurium in Guangdong after 2008. Meanwhile, a worrying percentage of multidrug resistant strains, including those resistant to quinolones and cephalosporin, were also observed in *Salmonella* Typhimurium [12]. However, the available routine surveillance methods that were used, including serotyping and pulsed-field gel electrophoresis (PFGE), are not suitable for reconstructing evolutionary paths of globally distributed clonal lineages in *Salmonella* Typhimurium. We therefore used a DNA sequence-based approach to investigate the population structure and potential evolutionary trends within *Salmonella* Typhimurium in Guangdong, China.

MLST is a DNA sequence-based method using multiple housekeeping genes which can reveal the genetic relatedness of bacterial strains and reconstruct their evolutionary paths. MLST was first developed by Maiden *et al.* in *Neisseria meningitidis* [13], and it has since been applied to many other bacterial species [14], [15], [16]. MLST has several advantages over non-sequence based methods, including PFGE, which rely on gel banding patterns. In particular, the DNA sequence is unambiguous, and data from different labs can easily be compared. MLST also allows the reconstruction of phylogenetic relationships within a population using a single combined character matrix with no loss of sequence information.

Several different MLST strategies have been examined in *Salmonella* isolated from the environment, animals or humans [17], [18], [19], [20]. We employed one of the schemes based on seven housekeeping genes that has been widely used and provides access to global data from a publicly accessible database (http://mlst.warwick.ac.uk/mlst/dbs/Senterica). We used this scheme to differentiate closely related *Salmonella* Typhimurium isolates into phylogenetically relevant clusters, to analyze the characters of nucleotide sequence data and genetic variation, and to explore the potential population structure and association with antimicrobial susceptibility profiles of *Salmonella* Typhimurium isolates in Guangdong, China.

## Materials and Methods

### Ethical standards

Ethical approval was granted by the Ethical Committee of Guangdong Provincial Center for Disease Control and Prevention. The present study complies with the World Health Organization and international guidelines on global surveillance. The procedure of sampling from outpatients is part of the standard diagnostic work-up of patients.

### Sampling, bacterial culture and identification

The sampling plan was previously described [12]. According to the Global *Salmonella* Surveillance program, the recruitment of patients followed specific guidelines. Patients who had two of the following three symptoms were chosen for the study: 1) diarrhea more than three times within 24 hours with watery stools. 2) fever >38°C, headache, chills and malaise. 3) diarrhea with vomiting, abdominal pain and watery stools. Stool samples were collected from clinical diarrhea outpatients of hospitals in fifteen cities from 2007 to 2011. All of the samples were cultured in local hospitals using solid broth agar media (Huankai, Guangzhou, China) for the initial evaluation of *Salmonella* growth, and then suspicious colonies were picked into vials containing semi-solid Rappaport Vassiliadis Medium (OXOID, France), and shipped to Guangdong Provincial Center for Disease Control and Prevention (Guangzhou, China) at room temperature, where they were incubated daily and checked for growth. The cultures that grew were Gram stained, subcultured and identified at the species level by standard biochemical methods, including the characteristic growth on Kligler Iron Agar (Huankai, Guangzhou, China), tests for urease, oxidase, β-galactosidase and indole production, and positive tests for lysine decarboxylase. The serovar of all the isolates was determined with commercial antiserum (Remel, Lenexa, Kansas, USA) following the Kauffmann-White scheme [21]. The outbreak isolates were collected separately, and all the strains collected as described above were considered epidemiologically unrelated. Age, gender andgeographic origin were recorded as part of the standard information present on the laboratory request forms. In addition, nineteen *Salmonella* Typhimurium strains isolated from 1977 to 2002 were included in this study (Table S1).

### Genomic DNA isolation, PCR amplification, sequencing and MLST

Genomic DNA was isolated using the InstaGene Matrix (BioRad, Hercules, USA) according the manufacturer’s protocol. In brief, colonies from overnight cultures were washed in water. InstaGene Matrix was added to the cells, and the mixture was incubated at 56°C for 30 min. After being vortexed vigorously for 10 s, the cells-matrix mixture was incubated at 100°C for 8 min and then centrifuged at 13,000 g for 10 min. The supernatants were collected and used for PCR templates. PCR reactions were prepared by combining 2 µl of isolated DNA with PCR buffer containing a final concentration of 1.5 mM MgCl_2_ (Applied BioSystems, Foster, USA), 0.2 mM of each dNTPs (Promega, Madison, USA), 0.2 mM of each of the appropriate forward and reverse primers, and 1.25 U of GoTaqDNA polymerase (Promega, Madison, USA). All the primer sequences for amplification and sequencing were obtained from the MLST Databases at the University of Warwick (www.mlst.warwick.ac.uk/mlst/dbs/Senterica). The PCR cycling conditions followed the online instructions. The PCR products were purified with Sephadex G-50 fine (GE Healthcare Bio-Sciences AB, Uppsala, Sweden). Nucleotide cycle-sequencing was performed directly on purified PCR templates using automated Sanger dideoxychain termination methods and the primers described on the MLST website. The sequences of seven housekeeping genes, *aroC*, *dnaN*, *hemD*, *hisD*, *purE*, *sucA* and *thrA*, were compared with the available MLST database (http://mlst.warwick.ac.uk/mlst/dbs/Senterica) to get the allele number and sequence typing (ST) number for each isolate. Sequence information of newly assigned alleles and STs were deposited in the MLST database.

### DNA polymorphic analysis and neutrality test

DNA polymorphism analysis, including the number of mutations, the number of alleles, the nucleotide diversity, the number of synonymous and nonsynonymous mutations, was carried out using DnaSPv5.10.00 software [22]. Tajima’s D test was used to compare the population mutation rate, which is estimated from the number of segregating sites, to the average pairwise nucleotide distance to determine whether the observed frequency of segregating mutations agreed with the expected frequency under the standard neutral model [23]. Allelic types and STs were assigned according to the MLST database of *Salmonella*.

### Phylogenetic reconstruction and clustering analyses

All of the sequences were edited using SeqMan software from the Lasergene software package (DNASTAR, USA). Iterative alignment was performed automatically with manual adjustment in BioNumerics 6.5 (Applied Maths, Belgium). A minimal spanning tree was generated from the allelic profiles of isolates using the predefined template in BioNumerics 6.5 designated as MST for categorical data, which preferentially joins single and double locus variants with the largest number of isolates per ST. All the reliable STs that belong to *Salmonella* Typhimurium on the website (http://mlst.warwick.ac.uk) were used for this tree construction (Table S2).

A dendrogram was built using MEGA software 5.0 [24] to determine the relationships among the different STs. Phylogenetic neighbor joining trees were inferred for concatenated sequences to determine the variable sites in seven loci using MEGA 5.0 software [24]. The percentage of replicate trees in which the associated taxa clustered together was estimated by a bootstrap test inferred from 1000 replicates. ClonalFrame [25] was used to infer their phylogenetic relatedness based on sequence alignments of gene fragments for one representative from each of these STs. ClonalFrame identifies regions that are likely to have arisen by homologous recombination and accounts for them when reconstructing the clonal genealogy. The lengths of the branches in the evolutionary tree are measured in coalescent time units, which are equal to the effective population size multiplied by the average duration of a generation. The nucleotide sequence alignments for all gene fragments from unique STs were used to infer clonal relationships with ClonalFrame from the 50% consensus of 10 runs, each with 100,000 iterations following a burn-in phase of 100,000 iterations.

### Antimicrobial susceptibility

The antimicrobial susceptibility test was performed according to the guidelines of the Clinical and Laboratory Standards Institute (CLSI) [26]. Agar diffusion assays were performed on Muller-Hinton agar with disks containing streptomycin, 10 µg (S), gentamicin, 10 µg (G), ceftazidime, 30 µg (Caz), cefotaxime, 30 µg (Ctx), cefepime, 30 µg (Fep), ampicillin, 10 µg (A), nalidixic acid, 30 µg (Nal), ciprofloxacin, 5 µg (Cp), tetracycline, 30 µg (T), chloramphenicol, 30 µg (C), sulfamethoxazole, 300 µg (Su), and trimethoprim, 5 µg (Tm), respectively. The results were interpreted according to the CLSI guidelines [26]. *Escherichia coli* ATCC 25922 was used as the quality control organism. Multi-resistance was identified as resistance to more than three classes of antimicrobials. The antimicrobial resistance pattern as well as the source of isolation of each strain is shown in Table S1.

## Results

### Representative assessment of Salmonella Typhimurium and MLST determination

According to the latest data, the frequency of human salmonelosis in China was 549 per 100,000 people in 2013 (China CDC surveillance system, unpublished data), over 33 times as high as human infections in the USA in 2012 (16.4 per 100,000) [27]. Based on this frequency, 549,000 people in Guangdong Province (total population size 100 million) are infected by *Salmonella* annually. Thirty percent of *Salmonella* isolates in Guangdong Province are of serovar Typhimurium [12], which corresponds to approximately 164,700 human infections per year. The 275 isolates from 2007–2011 that are described here represent 0.0334% (275/164,700*5) of the estimated number of infections over five years.

We performed MLST sequencing of seven housekeeping genes on all 275 isolates as well as on 19 other *Salmonella* Typhimurium strains which were isolated between 1977 and 2002, for a total of 294 isolates (Table S1). We found only one *hemD* nucleotide sequence (allele) but multiple alleles in *aroC* (2 alleles), *dnaN* (4), *hisD* (3), *purE* (3), *sucA* (3) and *thrA* (6), of which one in each of *purE*, *sucA* and *thrA* was novel. The aligned sequences of the concatenated loci were 3,336 base pairs long, with 59 polymorphic sites (58 parsimony informative and 1 singleton sites). The seven loci yielded 13 Sequence types (STs), of which ten (11 isolates) were novel (Table 1). Most of the isolates were assigned to the previously defined ST34 (209, 71.1%) and ST19 (69, 23.5%) (Table 1). ST36 and ST1782 contained 5 and 2 other isolates, respectively, and one isolate was identified for each of the other nine STs.

**Table 1:** Polymorphism summary and tests for neutral evolution in each locus of the Guangdong isolates and the global set of strains.

Parameters	Phylogenetic Marker (a/b)								
	*aroC*	*dnaN*	*hemD*	*hisD*	*purE*	*sucA*		*thrA*	Concatenated loci
No. ofsequences	294/1186	294/1186	294/1186	294/1186	294/1186	294/1186		294/1186	294/1186
No. ofcharacters	501/501	501/501	432/432	501/501	399/399	501/501		501/501	3336/3336
No. ofcodons	167/167	167/167	144/144	167/167	133/133	167/167		167/167	1112/1112
	**DNA polymorphism** **analysis (a/b)**								
No. ofmutations (η)	5/14	9/10	0/9	15/23	4/11		5/14	21/29	59/110
synonymouschanges	5/13	7/7	0/3	13/17	3/8		4/11	21/28	53/87
nonsynonymouschanges	0/1	2/3	0/6	2/6	1/3		1/3	0/1	6/23
nucleotideVariability	0.998%/2.794%	1.796%/1.996%	0/2.083%	2.994%/4.591%	1.002%/2.757%		0.998%/2.794%	4.191%/5.788%	0.177%/3.297%
amino acidVariability	0/0.599%	1.197%/1.796%	0/4.166%	1.197%/3.593%	0.752%/2.256%		0.598%/1.796%	0/0.598%	0.539%/2.068%
nucleotidediversity (Pi)	0.00047/0.00113	0.00125/0.00145	0/0.00033	0.00026/0.00013	0.00012/0.00070		0.00039/0.00076	0.00087/0.00130	0.0005/0.00084
allele	2/6	4/5	1/5	3/9	3 (1)/9		3 (1)/11	6 (1)/15	13 (10)/47
	**Neutrality test (a/b)**								
Tajima’s D	−1.29146 (p>0.10)/−1.47225 (p>0.10)	−1.22590 (p>0.10)/−0.86813 (p>0.10)	na/−1.67213(0.10>p>0.05)	−2.31354(p<0.01)/−2.30983 (p<0.05)	−1.56342 (0.10>p>0.05)/−1.62052(0.10>p>0.05)		−1.38243(p>0.10)/−1.69198 (0.10>p>0.05)	−2.26094 (p<0.01)/−2.05595 (p<0.05)	−2.40175 (p<0.01)/−2.23945 (p<0.01)

a/b: the Guangdong isolates/the Global set of isolates.

**Figure 1:**
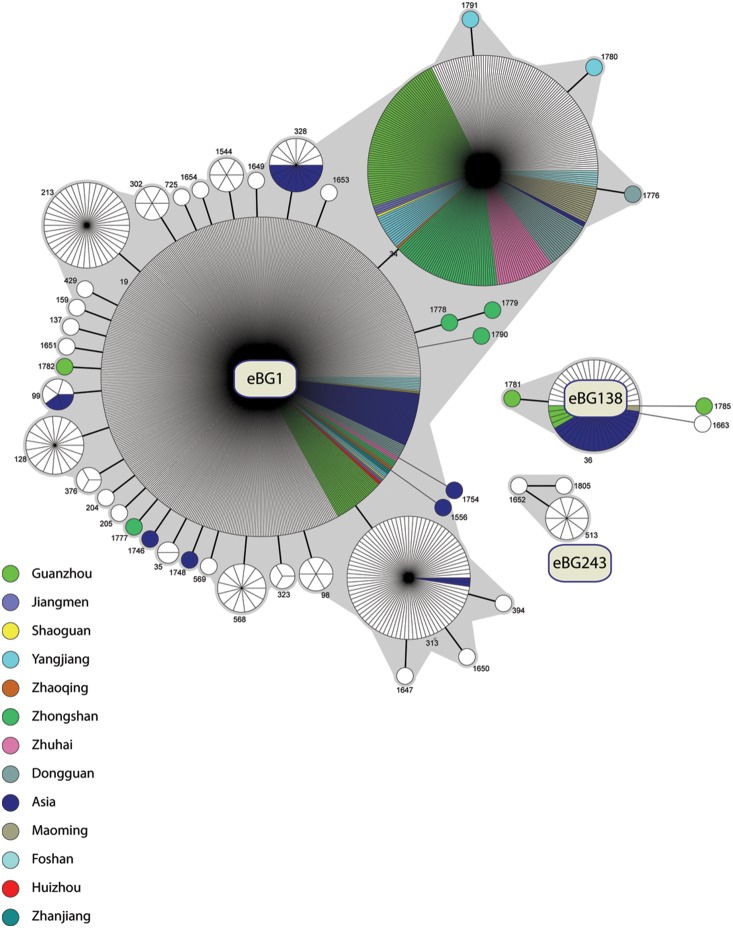
minimal spanning tree (MSTree) of the MLST data of *S. enterica* serovar Typhimurium. Each circle corresponds to each STs with the size proportional to the number of isolates. The topological arrangement within the MSTree is dictated by its graphic algorithm, which uses an iterative network approach to identify sequential links of increasing distance beginning with central STs that contain the largest numbers of isolates. As a result, singleton STs are scattered throughout the MSTree proximal to the first node that was encountered with shared alleles, even if equal levels of identity to other nodes that are distant within the MSTree exist. The figure only shows the links of six identical gene fragments (SLVs, thick black line) and five identical gene fragments (DLVs, thin black line) because these correlate with eBGs, which are indicated by grey shading. The key shows the isolates from Guangdong or Asia.

Achtman *et al.* [17] grouped *Salmonella enterica* STs into discrete eBGs (eBurstGroups), each of which includes two or more STs that are connected by pair-wise identities for six of the seven gene fragments, i.e., they share six of the seven alleles that define the ST. In other bacterial species, these groups are also referred to as “Clonal Complex” or “ST Complex”. We extended these groupings to include the ten novel STs described here as well as novel Typhimurium STs that have recently been uploaded to the MLST website by other scientists (Fig. 1). All Typhimurium STs fall into three eBURST groups (eBG1, eBG138 and eBG243), except for STs 1665 and 1783, which are double locus variants of eBG138. Most of the novel STs described in this study belong to eBG1 (ST1791, 1780, 1776, 1782, 1777), but ST1781 was assigned to eBG138. We did not find any isolates in eBG243, which is generally rare, and has not yet been isolated from Asia. STs 19 and 34, the two most common STs among the isolates from Guangdong province, are the two most commonly isolated STs at the global level and belong to eBG1. The most common ST in eBG138 is ST36 (Fig. 1).

**Figure 2:**
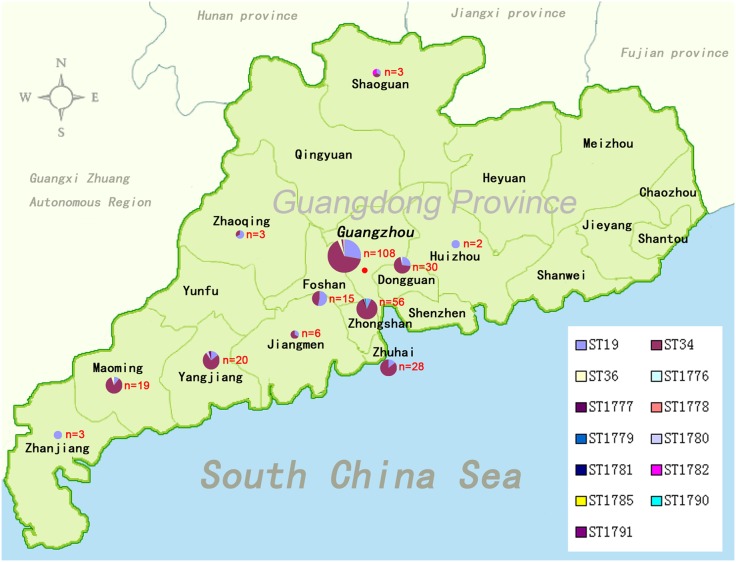
The locations where clinical diarrhea outpatients were collected and the STs assigned in each location in this study. Field sites are shown in relation to the ranges of the 12 proposed cities in Guangdong Province, China. The STs in each city are marked with red.

Comparative Geographical analysis showed that most isolates from Guangdong belong to ST34 within eBG1, rather than ST19, which is more common outside Asia (Fig. 2). The novel STs in eBG1 were ST 1776–1780, 1782, and 1790–1791. ST1781 belongs to eBG138, and ST1785 is a double locus variant of that eBG. All 19 isolates collected between 1977 and 2002 were assigned to ST19, and all 11 isolates assigned to novel STs were isolated after 2007 (Fig. 2).

### Descriptive analysis of nucleotide sequence data

The nucleotide diversity of DNA sequences in the isolates from Guangdong ranged from 0 for *hemD* to 0.00125 for *dnaN*, corresponding to between 0 and 4.191% of the nucleotides being variable in each locus. The amino acid variability ranged from 0–1.197% due to two variable amino acids in *dnaN* and *hisD* and one each in *purE* and *sucA* (Table 1). Both the nucleotide diversity and the amino acid variability in the global set of isolates showed the same trend, but with higher values than in Guangdong isolate (Table 1). The nonsynonymous and synonymous base substitution and amino acid variability were summarized in Table 2.

**Table 2:** Synonymous and non-synonymous changes in DNA and amino acid sequence in seven genes of *Salmonella* serovar *Typhimurium* in this study.

Gene	Total codons	1^st^ base	2^nd^ base	3^rd^ base	Amino acid change	Strains
*aroC*	167			14	ATG→ATA/M→I	ST328
*dnaN*	167	3		7	GGC→AGC/G→S	ST1653
					TCG→GCG/S→A	ST36, ST1663
					GTG→ATG/V→M	ST34
*hemD*	144	1	2	3	GCG→ACG/S→T	ST1754
					GTG→GCG/V→S	ST513, ST1652, ST1754, ST1805
					GCG→TCG/S→A	ST513, ST1652, ST1754, ST1805
					GCG→GTG/S→V	ST98
					CGC→CAC/R→H	ST513, ST1652, ST1805
					CGC→CAC/R→H	ST1663
*hisD*	167	2	3	18	AAG→AAC/K→N	ST1754, 1785, 1790
					GGC→GAC/G→D	ST1790
					GCG→GAG/S→E	ST1663
					CCG→CTG/P→L	ST394
					GAT→AAT/D→N	ST1649
					GTC→ATC/V→I	ST1544
*purE*	133		1	3	GTA→ATA/V→I	ST213
					GCC→GAC/A→D	ST1777
					CAG→GAG/Q→E	ST302, 313
*sucA*	167	4	1	9	ACC→ATC/T→I	ST1780
					GGC→TGC/G→C	ST137
					GTG→CTG/V→L	ST128
*thrA*	167	2		27	ACG→TCG/T→A	ST99
Total	787	12	7	81		

A, Alanine; C, Cysteine; D, Ariginine; E, Glutamic acid; G, Glycine; H, Histidine; I, Isoleucine; K, Lysine; L, Leucine; M, Methionine; N, Asparagine; P, Proline; Q, Glutamine; R, Argnine; S, Serine; T, Threonine; V,Valine.

Tajima’s D test for the data set showed positive selection in *hisD* and t*hrA* genes (p<0.05), but was rejected in the other genes in the isolates from Guangdong as well as in the global set of isolates (p>0.05) (Table 1). The neutrality evolution test within each eBG using the global set of isolates showed positive selection in *aroC*, *hemD*, *hisD*, *sucA* and t*hrA* genes (p<0.05) in eBG1 as well as in *hisD* and *thrA* (p<0.05) in eBG138, but was rejected in eBG243 (p>0.05) (Table 3).

**Table 3:** A neutrality test of three eBGs on seven loci in the global strains from MLST database.

	eBG1 (ST19, 34, 1776–80,1782, 1790–91) (n = 1123)	eBG138 (ST36,1781, 1785) (n = 51)	eBG243 (ST513, 1805, 1652) (n = 12)
*aroC* (501 bp)	−1.86501 (p<0.05)	0	0
*dnaN* (501 bp)	−0.97769 (p>0.10)	0	0
*hemD* (432 bp)	−1.72921 (p<0.05)	−1.10080 (p>0.10)	0
*hisD* (501 bp)	−2.23874 (p<0.01)	−2.29677 (p<0.01)	0
*purE* (399 bp)	−1.53932 (0.10>p>0.05)	0	0
*sucA* (501 bp)	−1.88897 (p<0.05)	0	0
*thrA* (501 bp)	−2.31093 (p<0.01)	−2.12375 (p<0.05)	−1.02689 (p>0.10)
Concatenated loci	−2.51802 (p<0.001)	−2.51435 (p<0.001)	−1.02689 (p>0.10)

### Phylogenetic analysis of the Salmonella serovar Typhimurium population

**Figure 3:**
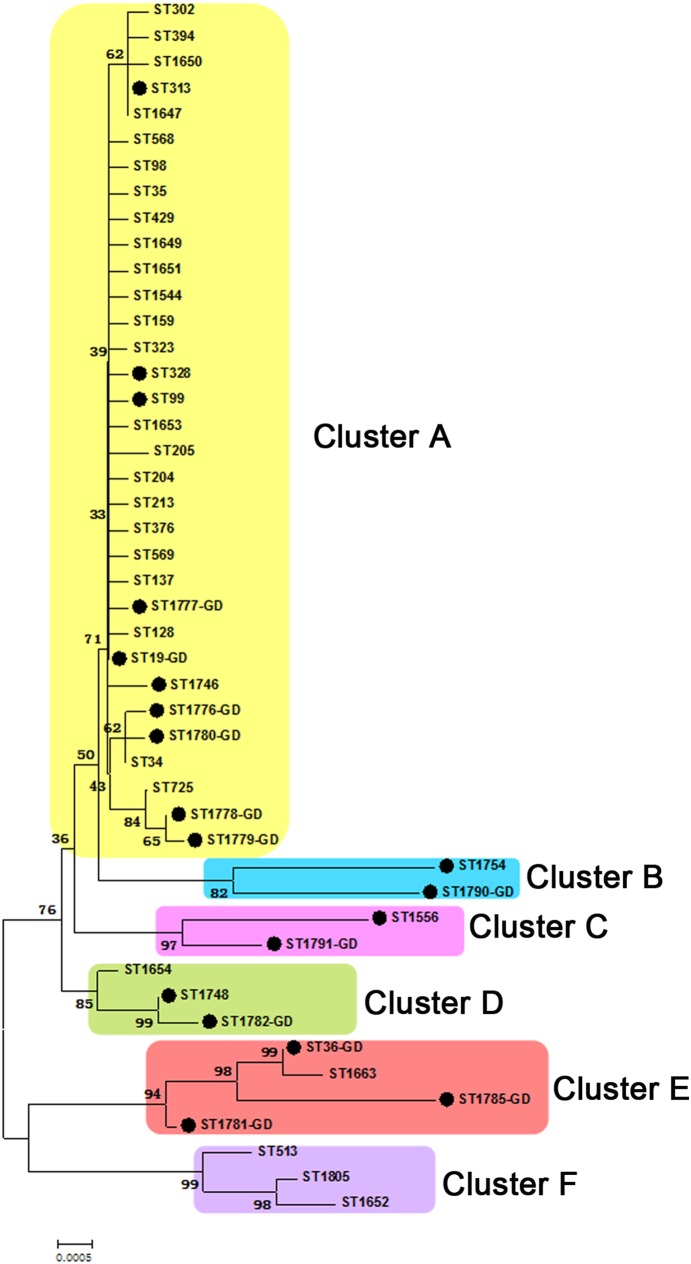
A neighbor joining tree showing the phylogenetic relationships of the *Salmonella* Typhimurium STs included in this study (n = 47). All STs were delineated into five major groups (bootstrap ≥70). The STs from Asia were labeled with a black dot in the phylogenetic tree, whereas STs from Guangdong were labeled GD. The percentages of replicate trees in which the associated taxa clustered together in the bootstrap test (1000 replications) are indicated. The evolutionary distances were computed using the neighbor joining method and are presented in units of the number of base substitutions per site.

**Figure 4:**
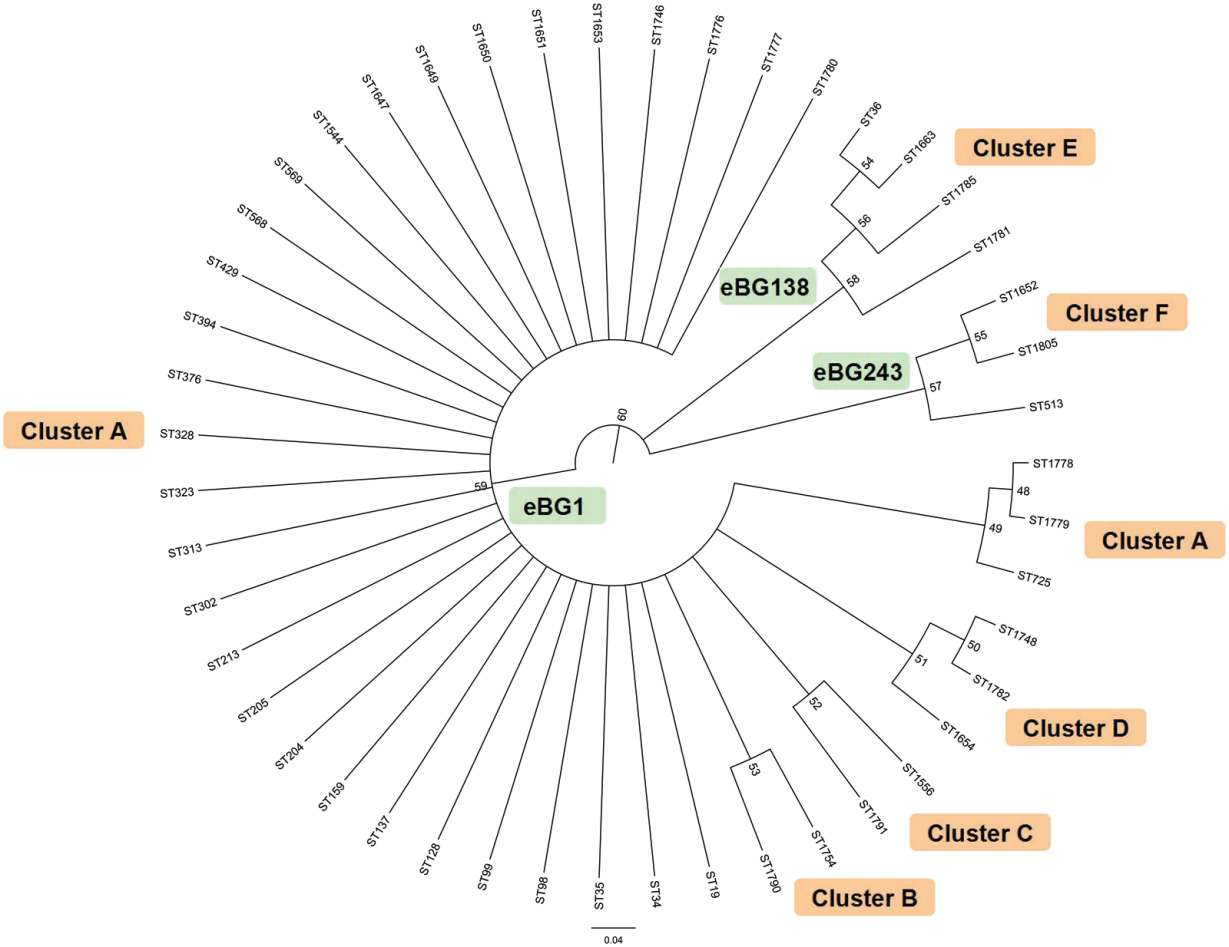
A consensus ClonalFrame tree constructed using ClonalFrame 1.1 in this study (ST = 47). The groups were marked using assigned clusters from the neighbor joining and minimal spanning trees.

The evolutionary relationships of the 294 *Salmonella* isolates tested in this study were determined with sequence information from the MLST database. A neighbor joining tree was generated for this purpose by concatenating sequences (3336 bp) from the unique STs (n = 47) (Fig. 3). The tree demonstrated that STs were clustered into six clusters (bootstrap ≥70), represented by A (yellow), B (blue), C (pink), D (green), E (red) and F (purple). Cluster A is composed of 7 STs from Guangdong, which covered most of the isolates from 12 cities. Clusters B–D are composed of 3 STs, including 4 isolates from Guangzhou, Zhongshan, Yangjiang and Shaoguan, whereas cluster E (3 STs, 7 isolates) was mostly found in Guangzhou (n = 6) and Maoming (n = 1). No STs from Guangdong were detected in cluster F (3 STs). However, we found that all the STs from Guangdong were closed to the STs originally observed in Asia (Fig. 3, black dot). According to the ClonalFrame analysis, *Salmonella* Typhimurium contains three groups, which correspond to eBG1, eBG138 and eBG243 on MStree (Fig. 4). The clusters B–D branch from the same root position within eBG1 as single STs in that eBG and a small cluster not recognized visually within the phylogenetic tree. These results indicate that much of the apparent phylogenetic clustering within eBG1 reflects recombination events in single locus. The estimated recombination ratio was 0.1691+/−0.0039 (rho/theta; frequency of recombination events per mutation event) resulting in 0.517+/−0.0098 (r/m) nucleotide replacements due to recombination rather than mutation.

### Antimicrobial susceptibility

A large burden of antibiotics resistance was found for ampicillin (87.75%), nalidixic acid (82.65%), sulfamethoxazole (89.79%), trimethoprim (71.43%), tetracycline (89.12%), gentamicin (65.31%), streptomycin (78.57%) and chloramphenicol (74.49%) in the isolates from Guangdong (Table 4), while less resistance in four antimicrobial agents consist of ceftazidime, cefotaxime, cefepime and ciprofloxacin (5.74%–13.39%). In particular, the resistance to cephalosporins is not infrequent observed. The isolates from ST 34 showed higher lever of resistance to all eleven antibiotics (except ciprofloxacin) than the isolates from ST 19. A few novel STs, including ST1778 and ST1790, showed high level of resistance to first or partial third or fourth generation antibiotics.

**Table 4:** Antimicrobial susceptibility and MDR among 294 *Salmonella enterica* isolates obtained from humans with infections in Guangdong province, China, between 2007 and 2011.

	No. ofisolates	ampicillin	ceftazidime	cefotaxime	cefepime	ciprofloxacin	nalidixicacid	Sulfamethoxazole	trimethoprim	tetracycline	gentamicin	streptomycin	chloramphenicol	MDR
ST34	209	202(96.65)	21(10.05)	28(13.39)	25(11.97)	12(5.74)	197(94.26)	204 (98.06)	163(77.99)	202(96.65)	157(75.12)	172(82.30)	178 (85.17)	206 (98.56)
ST19	69	44(63.77)	2(2.89)	6(8.69)	4(5.79)	10(14.49)	36(52.17)	46 (66.67)	37(53.62)	46(66.66)	28(40.58)	47(68.12)	31(30.55)	49(71.01)
ST36	5	3(60.00)	1 (20.00)	2 (40.00)	0	1 (20.00)	2(40.00)	4 (80.00)	2(40.00)	4(80.00)	1(20.00)	4(80.00)	3(60.00)	4 (80.00)
ST1776	1	1	0	0	0	0	1	1	1	1	1	1	1	1
ST1777	1	0	0	0	0	0	0	0	0	0	0	0	0	0
ST1778	1	1	0	1	1	0	0	1	1	1	0	0	1	1
ST1779	1	1	0	0	0	0	1	1	1	1	1	1	1	1
ST1780	1	1	0	0	0	0	1	1	1	1	1	1	1	1
ST1781	1	1	0	0	0	0	1	1	1	1	1	1	1	1
ST1782	2	2	0	0	0	1	2	2	2	2	1	2	1	1
ST1785	1	0	0	0	0	0	1	1	0	1	0	1	0	1
ST1790	1	1	1	1	1	0	1	1	1	1	1	0	1	1
ST1791	1	1	0	0	0	0	0	1	0	1	0	1	0	1
Total(% resistance)^b^	294	258(87.75)	25(8.50)	38(12.93)	32(10.88)	24(8.16)	243(82.65)	264(89.79)	210(71.43)	262(89.12)	192(65.31)	231(78.57)	219(74.49)	268(91.16)

Number of isolates resistant to indicated agent at the indicated breakpoint (% resistance)*^a^*.

a The percentage means the proportion of resistant isolates for each antibiotics.

b The percentage means the proportion of MDR isolates among the total isolates.

Multidrug resistance occurred in 91.16% (268/294) of the isolates. A large proportion of the MDR isolates were observed in ST 34 (98.56%) and in most of the novel STs (Table 4). The antimicrobial resistance patterns are shown in Table 4. In total, 57 resistant profiles were observed, of which 9 resistant profiles (more than 5 strains in each profile, corresponding to 5 STs including ST34, 19 and 36) accounted for 65.64% (n = 193) of all isolates (Table 5). The resistant profiles, including ACSSuTTmNaG, ACSSuTTmNa, ACSuTTmNaG, and ASSuTNa were found to be specific for ST34 and ST19. ACSSuT, the typical resistance pattern for Salmonella Typhimurium, was found in 179 isolates (66.79%). Three *Salmonella* Typhimurium isolates resistant to all twelve antimicrobial agents were obtained (ESS221, ESS463 and ESS797). Only four isolates were fully susceptible to all the agents tested (Table S1).

**Table 5:** *Salmonella* Typhimurium resistance type and the corresponding MLST type in this study.

	Mainly MDR Type (N, %)^a^	MLST Type (n/N, %)^b^
Typical pattern	ACSSuT (179, 66.79%)	ST34 (146, 81.56%)
		ST19 (25, 13.97%)
		ST36 (3, 1.68)
1	ACSSuTTmNaG (107, 39.93%)	ST34 (97, 90.65%)
		ST19 (6, 5.61%)
2	ACSSuTTmNa (17, 6.34%)	ST34 (14, 82.35%)
		ST19 (3, 17.65%)
3	ACSuTTmNaG (13, 4.85%)	ST34 (13, 100%)
4	ASSuTNa (13, 4.85%)	ST34 (11, 84.62%)
		ST19 (1, 7.69%)
5	ACSSuTTmNaGCp (12, 4.48%)	ST34 (6, 50%)
		ST19 (6, 50%)
6	ACSSuTNaG (11, 4.11%)	ST34 (10, 90.90%)
		ST19 (1, 9.10%)
7	ACSSuTTmNaGCazCtxFep (8, 2.99%)	ST34 (8, 100%)
8	ACSSuTTm (7, 2.61%)	ST34 (5, 71.43%)
		ST19 (1, 14.29%)
		ST36 (1, 14.29%)
9	ASSuT (5, 1.87%)	ST34 (5, 100%)
Total^c^	193/294, 65.64%	ST34 (169/268, 63.06%)
		ST19 (18/268, 6.72%)
		ST36 (1/268, 0.37%)

a Resistant pattern of *Salmonella* Typhimurium occurring in at least 5 strains. N = total number of isolates with the specific resistant pattern, % = hundred percentage in all isolates.

b MLST type that includes more than 2 strains. n = number of isolates occurring in the present STs, N = total number of isolates involving the present resistant pattern, % = hundred percentage in this ST.

c The proportion of MDR profile isolates among the total isolates, as well as in each ST.

## Discussion

Sequence analysis of all the isolates found that the most common STs observed were ST19 and ST34 in 12 cities of Guangdong province. The comparison of these data with the MLST database indicated that these two STs are relatively common in a range of hosts, including poultry, soya, fishmeal, lizards and humans (http://www.mlst.net). ST19 and ST34 were previously defined as a unique eBG1 by Achtman *et al.* [17], thus eBG1 was the predominant group in Guangdong, China. The archived information from the MLST database showed that the most prevalent ST in eBG1 is ST19. Unusually, more of the isolates from Guangdong were ST34 rather than ST19. Marcus *et al*. [8] showed that ST34 was also the major MDR ST of *Salmonella* Typhimurium in Hongkong, China. Therefore, one possibility is that ST34 is mainly endemic in Asia, whereas ST19 seems to be globally distributed. Within eBG1, there are common STs found elsewhere but not in Asia, including ST213, 128, 568, and ST313 which was the predominant ST in Africa but has spread globally. However, we did not find any isolates in ST313 and its descendants, ST1647, 1650, 394, or in ST328 and ST99, which are common in Taiwan and elsewhere. These observations were unexpected given the increasing cargo trade and frequent human travel between Guangdong and other countries. However, we did find STs in eBG138 that have been previously isolated in all continents, particularly in Taiwan, Japan, Malaysia and Thailand of Asia, suggesting that there are geographic specificities for the prevalence of different STs, possibly reflecting different intensities of the trade of food products with countries within Asia and elsewhere, or different dynamics in the spread of individual STs. Food associated with domestic animals (e.g., pork, chicken, egg and milk) are considered as the primary source of infection in Guangdong [12]. However, recent results have indicated that Typhimurium infection in humans is associated with clades distinct from those common in domesticated animals [3]. Therefore, it can be speculated that the transmission of the major eBGs or STs is a mixture spreading due to human travel and international trade in food products between different continents [28], [29], [30].

Searching the allele profiles of the MLST international database for *Salmonella*, we found that most of the novel STs are SLVs of either ST19 or ST34, which reflect a mixture of recombination and mutation, consistent with previous conclusions for the serovar Newport [20], [31]. Similar trends were observed for ST36 and its descendent STs. These data indicated that these strains probably displayed divergent evolution. ST19 and ST34 were the predominant clone lineage over time, and the novel STs were expanded from this lineage by a recent genetic event (mutation or recombination). Tajima’s D test, an effective tool for making inferences about population demographics [23], supports the hypothesis that ecological adaptation or slight geographic expansion occurred within some of the STs (Table 1). That is because the test values were significantly different from zero for all seven loci (p<0.01) and for two individual loci in the isolates from Guangdong (*hisD* and *thrA*, p<0.05). In addition, Tajima’s test within each eBG that potentially share a recent common ancestor, also showed positive selection in *hisD* and *thrA* in a global set of isolates (p<0.05). However, Zhou *et al.* claim that in *Salmonella* Agona [32] most genetic changes are due to changes of bacterial phages and other accessory genes, which result in changing the PFGE pattern but not the phenotype. The similar study by Zhou *et al.* in *Salmonella* Paratyphi A [33] showed that evolution is not adaptive but neutral due to transient Darwinian evolution; thus adaptations for particular ecological niches at the level of STs are rare. Indeed, we found a high estimated recombination ratio in the ClonalFrame tree, which means that the nucleotide replacements are due to recombination rather than mutation. However, greater genomic resolution will be needed to address such adaptation within *Salmonella* Typhimurium in a future study. Phylogenetic trees constructed by concatenating sequences from all the STs of *Salmonella* Typhimurium in MLST database (n = 47) showed that all STs were separated into six clusters, which correspond to three clades in the Clonalframe tree, whereas previous phylogenetic analyses by Sangal *et al.* [31] indicated that all Typhimurium belonged to a single clade with the exception of ST36, which was closely related to that clade. In this study, a much larger number of bacterial isolates and many more STs contributed to the result of three related clades, corresponding to both neighbor joining (Fig. 3) and the ClonalFrame tree (Fig. 4).

Synonymous and nonsynonymous substitutions were found in coding regions. There are 95 synonymous and 6 nonsynonymous substitutions in the isolates from Guangdong, whereas many more substitutions were found in a global set of isolates. One coding frame shift mutation was detected in *hisD*. Nonsynonymous substitutions was also detected in previous studies of *Salmonella enterica* [34], and is attributed to the threshold for slightly deleterious mutations within clones of a species [35]. However, a more traditional explanation of nonsynonymous substitutions is that the genes concerned were changing under selection pressure. A similar finding reported by Feil *et al*. [36] in a study of *Staphylococcus aureus* showed that a higher proportion of nonsynonymous substitutions occurred in closely related isolates that at the species or higher taxon levels. Nevertheless, the nonsynonymous substitutions within the one serovar of *Salmonella enterica* examined in this study will be worthy of further investigation.

The previous studies of salmonellosis in Guangdong illustrated the emergence of antimicrobial resistance in local populations; however, the genotype could not account for the observed phenotype [37], [38], [39]. Using MLST in this study, we demonstrated a higher proportion of MDR in ST19 and ST34, which were the most common STs in Guangdong. In particular, ST34 has been proved to be the predominant ST carrying multiple resistance determinants in Hongkong, China [8]. In addition, a few novel STs, including ST1778 and ST1790, showed high level of resistance to all the antibiotics tested. Therefore, further studies to determine the nature of resistance to related antibiotics are ongoing, whereas mobile genetic elements, including integrons, plasmids, genomic islands and transposons are possibly responsible for the observed resistance.

In conclusion, we presented the genotypic characterization of 294 *Salmonella* isolates from Guangdong, China, and used MLST to compare these isolates with a global set of isolates of this serovar. The 294 isolates were assigned to 13 STs, of which 10 were novel. All STs were further assigned to two eBurst Groups, eBG1 and eBG138. The genetic diversity analysis suggested a likely slight genetic selection within *Salmonella* Typhimurium population. Resistant profiles specific for particular STs were observed. Further evaluations with additional isolates obtained from various sources and regions are needed to reveal the scope of the epidemiological characteristics of *Salmonella* Typhimurium in Guangdong, China.

## Supporting Information

Table S1 **The** ***Salmonella*** **Typhimurium strain information isolated from 1977 to 2002 in this study (Table S1).**
(XLS)

Table S2 **The information of all the reliable STs of** ***Salmonella*** **Typhimurium on the website (** http://mlst.warwick.ac.uk **).**
(XLS)
